# Neuroprotection in radiotherapy of brain metastases: A pattern-of-care analysis in Germany, Austria and Switzerland by the German Society for radiation Oncology − working group Neuro-Radio-Oncology (DEGRO AG-NRO)

**DOI:** 10.1016/j.ctro.2024.100783

**Published:** 2024-04-20

**Authors:** N. Gleim, A. Rühle, S. Heider, F. Nägler, F.A. Giordano, S.E. Combs, J. Becker, M. Niyazi, A.L. Grosu, N.H. Nicolay, C. Seidel

**Affiliations:** a Department of Radiotherapy and Radiation Oncology, University Hospital Leipzig, Stephanstraße 9a, Leipzig, Germany; b Department of Radiation Oncology, University Medical Center Mannheim, Theodor-Kutzer-Ufer 1-3, Mannheim, Germany; c DKFZ Hector Cancer Institute, Theodor-Kutzer-Ufer 1-3, Mannheim, Germany; d German Cancer Research Center (DKFZ), Im Neuenheimer Feld 280, Heidelberg, Germany; e Mannheim Institute for Intelligent Systems in Medicine (MIiSM), Theodor-Kutzer-Ufer 1-3, Mannheim, Germany; f Department of Radiation Oncology, Klinikum Rechts der Isar, School of Medicine, Technical University of Munich, Ismaninger Straße 22, Munich, Germany; g Department of Radiotherapy and Special Oncology, Medizinische Hochschule Hannover, Carl-Neuberg-Straße 1, Hannover, Germany; h Department of Radiation Oncology, University Hospital Tübingen, Hoppe-Seyler-Straße 3, Tübingen, Germany; i Center for Neuro-Oncology, Comprehensive Cancer Center Tübingen-Stuttgart, University Hospital Tübingen, Herrenbergerstraße 23, Tübingen, Germany; j German Cancer Consortium (DKTK), Partner Site Tübingen, A Partnership between DKFZ and University Hospital Tübingen, Auf der Morgenstelle 15, Tübingen, Germany; k Department of Radiation Oncology, University of Freiburg - Medical Center, Robert-Koch-Straße 3, Freiburg, Germany; l Comprehensive Cancer Center Central Germany, Partner Site Leipzig, Liebigstraße 22, Leipzig, Germany

**Keywords:** Brain metastases, Radiotherapy, Neuroprotection, Hippocampal sparing, Memantine

## Abstract

**Background and purpose:**

Many patients with solid tumors develop brain metastases (BM). With more patients surviving long-term, preservation of neurocognitive function gains importance. In recent years, several methods to delay cognitive deterioration have been tested in clinical trials. However, knowledge on the extent to which these neuroprotective strategies have been implemented in clinical practice is missing.

**Materials and methods:**

We performed an online survey regarding treatment patterns of BM in German-speaking countries, focused on the use of neuroprotective approaches. The survey was distributed among radiation oncologists (ROs) registered within the database of the German Society for Radiation Oncology (DEGRO).

**Results:**

Physicians of 78 centers participated in the survey. Whole brain radiotherapy (WBRT) is still preferred by 70 % of ROs over stereotactic radiotherapy (SRT) in patients with 6–10 BM. For 4–5 BM WBRT is preferred by 23 % of ROs. The fraction of ROs using hippocampal sparing (HS) in WBRT has increased to 89 %, although the technique is used on a regular basis only by a minority (26 %). The drug memantine is not widely prescribed (14% of ROs). A trend was observed for university hospitals to implement neuroprotective approaches more frequently.

**Conclusion:**

There is considerable heterogeneity regarding the treatment of BM in German-speaking countries and a general standard of care is lacking. Neuroprotective strategies are not yet standard approaches in daily clinical routine, although usage is increasing. Further clinical trials, as well as improvement of technical opportunities and reimbursement, might further shift the treatment landscape towards neuroprotective radiation treatments in the future.

## Highlights

• Treatment patterns in radiotherapy of BM vary greatly in German-speaking countries.
• Whole brain radiotherapy (WBRT) is still often applied in patients with ≥4 BM.
• Neuroprotective approaches are not yet standard procedures in clinical routine.
• Use of hippocampal-sparing techniques is increasing.
• The NMDA antagonist memantine is rarely prescribed together with WBRT.

## 1 Introduction

Brain metastases (BM) constitute a common complication in patients with solid tumors, occurring in 10–40 % of all patients [1], [2]. Quality of life and delaying neurocognitive decline is of major importance for patients with BM, particularly for the increasing fraction of patients with long-term survival. In the past, whole brain radiotherapy (WBRT) was commonly used as a mainstay of treatment for brain metastases independent of their localization or number, but the role of WBRT has been diminished due to an association with frequent neurocognitive decline [3], [4], [5]. In specific entities such as non-small cell lung cancer (NSCLC), limited efficacy and the availability of targeted therapies have further contributed to this trend [6], [7]. In recent years, there has been increasing evidence for the efficacy of neuroprotective measures in radiotherapy of BM.

First, through anatomic sparing of unaffected brain structures, stereotactic radiotherapy (SRT) effectively preserves neurocognitive function compared to WBRT and can be used instead of WBRT in patients with up to 10 BM and potentially more without compromising survival [8], [9]. More recently, hippocampal-sparing whole brain radiotherapy (HS-WBRT) has been demonstrated to better maintain cognitive function compared to conventional WBRT in phase II and III trials [10], [11], [12]. Concerning potential neuroprotective drugs, the NMDA receptor antagonist memantine has shown to reduce radiation-induced neurotoxicity in a large phase III trial, albeit failing its primary endpoint [13], potentially due to unexpectedly high dropout rates. In a second phase III trial, HS-WBRT together with memantine was superior to conventional WBRT and memantine regarding several cognitive endpoints [11]. However, some aspects of these studies, such as relevant endpoints and confounding factors, have been subject to discussion [14], [15], [16]. As a result, varying guidelines and panel recommendations exist, providing a heterogeneous basis for the treatment landscape of BM.

While treatment patterns are shifting in the USA based on the published trial data [17], it is largely unknown to which extent neuroprotective strategies have been implemented in the therapeutic landscape of BM and the current daily treatment practice in Europe. In order to shed some light on this poorly analyzed area, we performed a survey among radiotherapy units in German-speaking countries (Germany, Austria and Switzerland) to analyze current practice patterns regarding the use of neuroprotective measures in the radiation treatment of BM.

## 2 Materials and methods

We compiled an online survey consisting of 29 questions regarding characteristics of treatment centers and expert radiation oncologists (ROs), as well as institutional standard operating procedures for BM treatment and follow-up, use of cognition tests and prognostic scores, and use of neuroprotective measures such as SRT, HS-WBRT and memantine. The survey was set up using SurveyMonkey® (SurveyMonkey Inc., San Mateo, California, USA) and was distributed within radiation oncologists registered within the German Society for Radiation Oncology (DEGRO, *Deutsche Gesellschaft für Radioonkologie*) on April 19, 2023. Two reminders were sent, each two weeks apart. Data collection was closed on June 5, 2023.

Data were collected centrally and analyzed using R Statistical Software version 4.3.1 [18]. Answers were restricted to one response from each center. Descriptive statistics were used to quantify all answers, χ2 tests or Fisher’s exact tests were used for subgroup analyses regarding the use of neuroprotection. Test results with p < 0.05 were considered statistically significant. The entire survey with all answers is available in Supplementary Table 1.

## 3 Results

### 3.1 Characteristics of radiotherapy units and responding physicians

The survey was completed by physicians of 78 centers (18.2 % of all invited centers), consisting of 24 university hospitals, 24 non-university hospitals and 30 outpatient centers for radiotherapy, each physician representing their center. Four responses were obtained from centers in Austria, four from centers in Switzerland and the remaining 70 from Germany. Centers treating 10–50 patients with BM per year formed the largest group (32 centers; 41.6 %), followed by centers with 51–100 (28; 36.4 %) and > 100 (17; 22.0 %) patients per year. More than half (41; 53.2 %) of responding ROs were employed in a leading position of their respective facility (Table 1).

**Table 1:** Basic demographic data, treatment and follow-up patterns, technical aspects of WBRT. Percentage values are shown relative to number of responding ROs. Abbreviations: RT radiotherapy, GPA graded prognostic assessment, dsGPA disease-specific GPA, RPA recursive partitioning analysis.

Basic demographic data	n	%
**Type of radiotherapy facility**		
University hospital	24	30.8 %
Non-university hospital	24	30.8 %
Outpatient center	30	38.4 %
**Country**		
Germany	70	89.8 %
Austria	4	5.1 %
Switzerland	4	5.1 %
**Treated patients with BM per year**		
< 10	0	0.0 %
10–50	32	41.6 %
51–100	28	36.4 %
> 100	17	22.0 %
**Leading position in RT facility**		
Yes	41	53.2 %
No	36	46.8 %

**Treatment and follow-up patterns**	**n**	**%**
**Imaging/Clinical follow-up organized by**		
Radiotherapy	37/44	48.1 %/57.1 %
Oncology	49/52	63.6 %/67.5 %
Neurology	3/1	3.9 %/1.3 %
Neurosurgery	11/12	14.3 %/15.6 %
Interdisciplinary	8/8	10.4 %/10.4 %
**Routine use of cognitive tests**		
Prior to radiotherapy	11	14.5 %
Upon completion of radiotherapy	1	1.3 %
During follow-up	11	14.5 %
None	53	69.7 %
**Use of prognostic scores regarded as**		
Very important	8	10.4 %
Important	33	42.9 %
Rather unimportant	30	38.9 %
Not important	6	7.8 %
**Used prognostic scores**		
GPA score	12	15.8 %
dsGPA score	11	14.5 %
RPA score	17	22.2 %
Other	3	3.9 %
None	33	43.4 %

**Technical aspects of WBRT**	**n**	**%**
**WBRT dose concept**		
3 Gy single, 30 Gy total dose	54	70.1 %
2 Gy single, 40 Gy total dose	5	6.5 %
4 Gy single, 20 Gy total dose	3	3.9 %
Alternative	15	19.5 %
**Use of boost to BM**		
Regularly	12	15.4 %
Frequently (≥50 % of cases)	26	33.3 %
Occasionally (<50 % of cases)	36	46.2 %
Never	4	5.1 %
**Criteria for boost together with WBRT**		
Size	66	89.2 %
General condition	56	75.7 %
Histology	44	59.5 %
Location	43	58.1 %
Age	25	33.8 %
Other	11	14.5 %
**Boost performed as**		
Simultaneous integrated boost	62	80.5 %
Sequential	27	35.1 %
Stereotactic	25	32.5 %
None	4	5.2 %

### 3.2 General treatment and follow-up patterns

Both imaging follow-up and clinical follow-up of BM were predominantly performed by the treating facility for radiotherapy and/or oncology. Tests to assess cognition were applied routinely only by a fraction of ROs (23; 30.3 %). Among these ROs, the Mini Mental Status Test (MMST) was the most frequently performed cognition test (21; 91.3 %). Prognostic scores for the indication of WBRT were regarded as important (41; 53.3 %) or rather unimportant (36; 46.7 %) by about half of ROs, respectively. Among the ROs using available prognostic scores (43; 56.6 %), the recursive partitioning analysis (RPA) score was most commonly employed, followed by the graded prognostic assessment (GPA) and the disease-specific GPA (dsGPA) score (Table 1).

### 3.3 Technical aspects of WBRT

For WBRT, the majority (54; 70.1 %) of ROs used a hypofractionated concept of 10 fractions of 3 Gy, with alternative fractionation concepts varying between 2–4 Gy (dose per fraction) and 20–42 Gy (total dose). A boost to individual BM as part of WBRT was performed at least occasionally by most ROs (74; 94.9 %), although only about half of ROs stated frequent or regular use (38; 48.7 %). Among the essential criteria to decide on a boost as part of WBRT, size, histology and location of the BM and the general condition of the patient were most frequently chosen. Simultaneous integrated boost concepts were employed more commonly (62; 80.5 %) than both sequential normo-/hypofractionated boost and stereotactic boost (27; 35.1 % and 25; 32.5 %) as method of choice, as shown in Table 1.

### 3.4 Application of neuroprotective radiotherapy strategies

#### 3.4.1 Stereotactic radiotherapy

**Fig.1:**
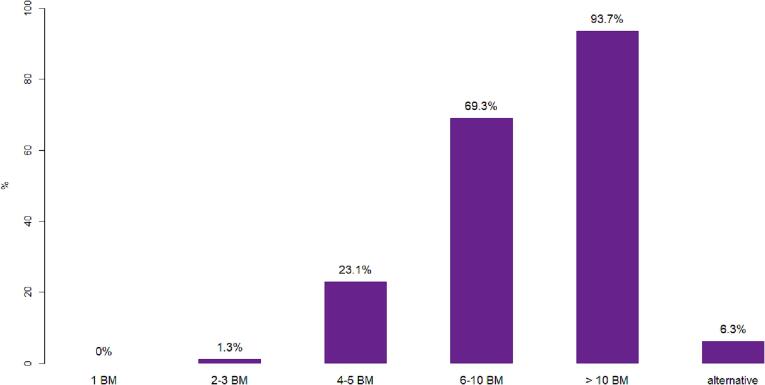
Application of WBRT versus SRT. Percentages of ROs preferring WBRT over SRT are shown for different numbers of BM in a patient.

Participating ROs were asked to report their institutional cut-off or preference for the use of SRT and WBRT regarding the number of BM. WBRT is routinely prescribed for 2–3 BM by 1.3 % of ROs (1), for 4–5 BM by 23.1 % (18), for 6–10 BM by 69.3 % (54) and for > 10 BM by 93.7 % of ROs (73) (Fig. 1). The remaining 6.3 % of ROs (5) stated flexible alternative decision strategies, with criteria such as BM volume, location and histology, RPA score, patient symptoms or general condition.

#### 3.4.2 Hippocampal-sparing WBRT

**Fig.2:**
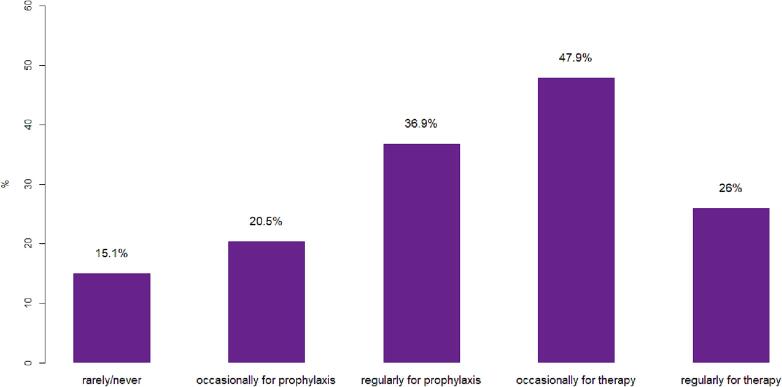
Application of HS-WBRT. Percentages of ROs using hippocampal sparing together with prophylactic or therapeutic WBRT with varying frequency are shown.

For patients with an indication for WBRT, most ROs (65; 89 %) stated that they use HS-WBRT at least occasionally, however, the frequencies of application varied with use in <10 % (25; 34.2 %), 10–49 % (23; 31.5 %), 50–80 % (12; 16.4 %) and >80 % (5; 6.8 %) of cases, respectively. Centers further differ in their use of HS-WBRT between therapy of BM and prophylactic treatment in small cell lung cancer (SCLC) (Fig. 2). For prophylaxis, regular use (27; 36.9 %) was stated more often than occasional use (15; 20.5 %). Conversely, more centers used HS-WBRT only occasionally (35; 47.9 %) than regularly (19; 26.0 %) for therapy. Reasons for not applying HS-WBRT included BM histology, visible adherent meningeosis foci (each 45; 63.3 %), estimated life expectancy less than 6 months (40; 56.3 %), Karnofsky performance score <60 % and multiple small BM without visible hippocampal involvement (each 33; 46.5 %), among others. Treatment doses implemented for HS-WBRT varied greatly between centers (Supplementary Table 1). In a scenario where one hippocampus was affected by metastasis, more than half of centers (42; 58.3 %) do not apply unilateral sparing of the unaffected hippocampus.

#### 3.4.3 Use of memantine

**Fig.3:**
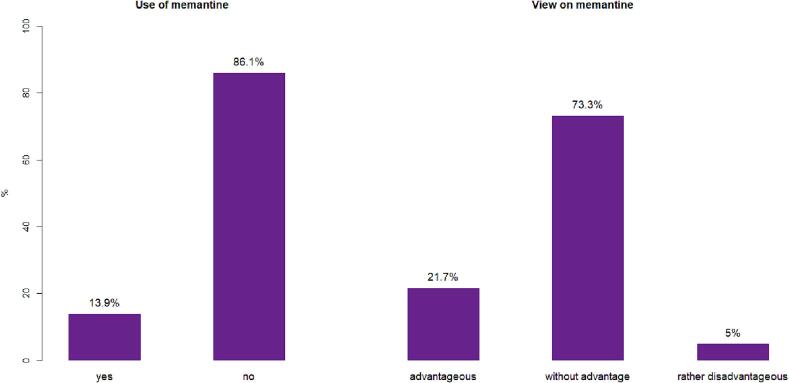
Usage of memantine (left) and view on the application together with WBRT (right).

As a concomitant treatment with WBRT, memantine is only prescribed by a minority of centers (10; 13.9 %) (Fig. 3). Memantine was infrequently used together with both prophylactic WBRT with and without concomitant hippocampal sparing (6; 8.6 % in HS-WBRT, 8; 11.3 % in WBRT), and together with therapeutic WBRT (9; 12.7 % in HS-WBRT, 10; 14.3 % in WBRT). Among the few ROs using concomitant memantine, the percentage of cases in which it was applied concomitantly to WBRT varied from < 10 % to > 80 % (details in Supplementary Table 1). The reimbursement process of memantine differed between prescription for self-payers (4; 40 %) and health insurance prescriptions (4; 40 %), as well as prescriptions by other physicians (2; 20 %). Most ROs (7; 70 %) using memantine prescribed it for a total duration of 6 months.

When questioned directly about their view on the use of memantine concomitantly to WBRT, only 5 % of ROs (3) regarded it as “rather disadvantageous”, compared to 73.3 % (44) seeing the use “without advantage” (Fig. 3). Interestingly, 21.7 % (13) of ROs considered memantine “advantageous”, indicating some discrepancy between the impression of an advantageous treatment and actual application in practice. The most common reason for not prescribing memantine was a lack of experience with the drug (30; 50.8 %), followed by the perception of insufficient available evidence (17; 28.8 %), potential problems with reimbursement for off-label use (10; 16.9 %) and concerns about side effects (2; 3.4 %).

### 3.5 Inter-facility differences in neuroprotection

**Fig.4:**
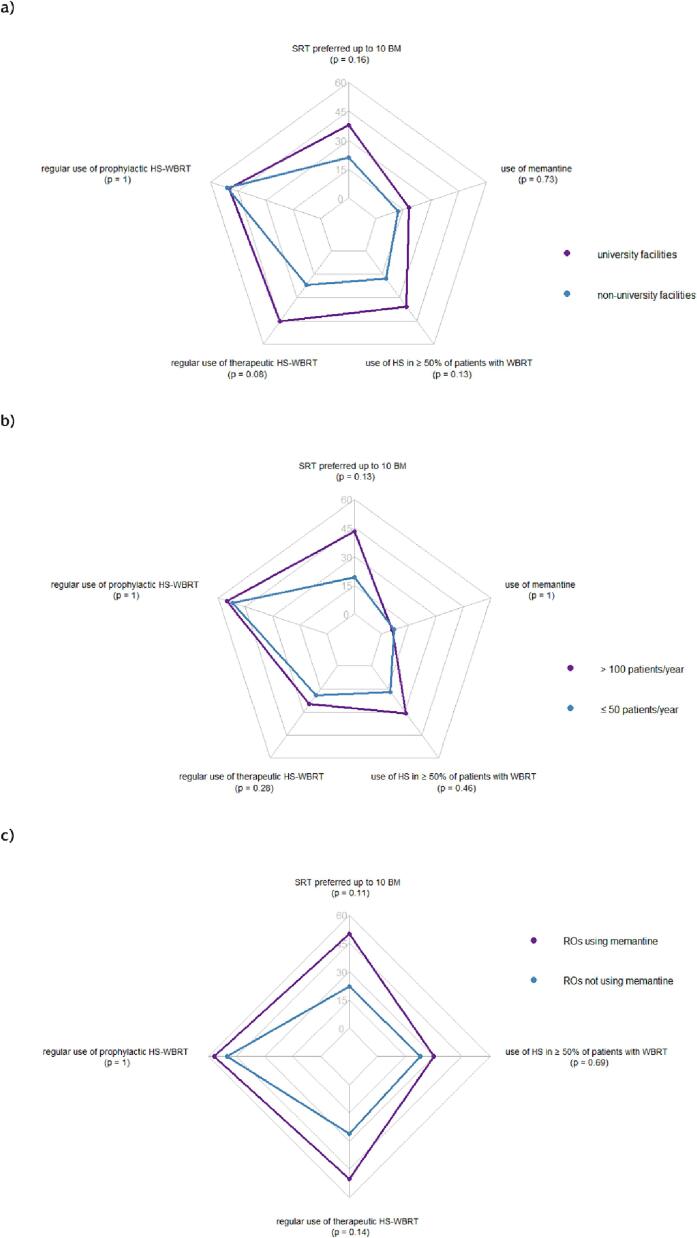
Differences in neuroprotective strategies between a) university hospitals (purple) and non-university medical facilities (blue), b) facilities treating >100 patients per year (purple) and facilities treating ≤50 patients per year (blue), c) ROs using memantine (purple) and ROs not using memantine (blue). Each respective question was binarized; percentages of positive answers on a scale from 0 to 60 % are shown for each subgroup.

The application of neuroprotective measures in the treatment of BM varied between different institution types. A trend could be observed for university hospitals to implement such measures more frequently (SRT for up to 10 BM [38 % vs. 21 %; p = 0.16], regular use of HS-WBRT for therapy [45 % vs. 22 %; p = 0.08], HS-WBRT in more than 50 % of patients [36 % vs. 18 %; p = 0.13]), as illustrated in Fig. 4a. An exception was the practice of HS-WBRT for prophylaxis (50 % vs. 51 %; p = 1), which was equally frequently applied in university and non-university hospitals.

In a comparison of facilities treating > 100 patients and facilities treating ≤ 50 patients per year, centers with many patients showed a trend for increased rates of using SRT for up to 10 BM (43 % vs. 19 %; p = 0.13), while HS-WBRT and memantine were equally frequently applied (Fig. 4b).

Among ROs not offering memantine in university hospitals, insufficient evidence was stated more often (42.1 %) as a reason for skepticism towards the drug than lack of experience with it (31.6 %). These points differed considerably with non-university facilities (22.5 % and 60.0 %, respectively), possibly reflecting different decision-making strategies in the academic setting.

Some concordance for the usage of different neuroprotective techniques was reported. For instance, ROs using concomitant memantine with WBRT also preferred SRT for a higher number of BM (50 % vs. 22 %; p = 0.11) and performed hippocampal sparing treatments more often (50 % vs. 26 %; p = 0.14), see Fig. 4c. Those ROs applying SRT for up to 10 BM also applied HS-WBRT more frequently for therapy. For both subgroups, no trend was observed regarding prophylactic HS-WBRT.

Further, Austrian and Swiss ROs prescribed SRT (57 % vs. 23 %; p = 0.08) and memantine (38 % vs. 12 %; p = 0.26) more often than German ROs, while HS-WBRT was less regularly applied.

## 4 Discussion

Within recent years, relevant efforts were made to improve radiotherapy of BM and to allow for better tolerance of treatment, e.g. by developing neuroprotective strategies. However, little is known about the actual usage of these strategies in patients with BM. In the US, several surveys show that the rates of ROs using memantine and HS-WBRT have been steadily increasing over the last 15 years. For instance, the percentage of ROs prescribing memantine for neuroprotection during WBRT has increased from 11 % to nearly 80 % between 2016 and 2022 and usage of HS-WBRT increased from 33 % to 73 % [17], [19], [20], [21]. Another recent retrospective analysis provided data from 8 different countries from Europe, Africa, Asia and North America, however restricted to patients receiving therapeutic WBRT [22]. Interesting patterns could be observed, such as substantial differences between North America and Europe for the use of memantine (25 % vs. 1.7 % of cases) and HS-WBRT (24 % vs. 4.6 %). Besides one German analysis executed in 2018 [23], to the best of our knowledge, further evidence on treatment patterns in Europe is lacking. Considering the enormous changes in the standard of care in the US over the last five years, new available data such as the NRG CC001 trial [11] and revised guidelines [7], [24], [25] have likely also altered the therapeutic landscape in German-speaking countries.

Our survey provides a comprehensive picture of the current use of radiotherapy for patients with BM. First, the regular use of prognostic scores has increased from 38 % in 2018 [23] to 57 %, with increasing popularity of the dsGPA score. Concerning the ongoing debate between WBRT and SRT, 70 % of ROs still preferred WBRT in patients with 6–10 BM, and, in contrast to several guideline recommendations, 23 % preferred WBRT even in patients with 4–5 BM. Nonetheless, the number of ROs using SRT for 5 and up to 10 BM is expected to further increase with increasing technological ease-of use. This possible form of neuroprotection might become more important in the future, as the incidence of multiple BM in patients is increasing [26]. While evidence for SRT for up to 10 BM has been known for a decade [8], evidence from a phase III clinical trial has recently been published for the feasibility of SRT up to 15 BM [9], also showing superior neurocognitive outcome compared to WBRT.

The percentage of ROs using HS-WBRT at least occasionally has increased from 56 % in 2018 [23] to 89 % in our analysis, which even exceeds the percentage of 73 % measured in the US [17]. Among those ROs using HS-WBRT, 26 % apply it to more than 50 % of their patients, slightly less than in the US (33 %), demonstrating that hippocampal sparing has not yet become a standard technique in clinical routine. The more frequent use in academic centers, which was also observed in the US, could point to resource or experience issues in this context. Surprisingly, in our analysis, a larger fraction of ROs stated frequent use of HS-WBRT for prophylactic cranial irradiation (PCI) in SCLC compared to BM therapy (37 % vs. 26 %), although there is less evidence for HS to reduce cognitive decline in this setting [27]. Additionally, the current EANO guidelines explicitly state that HS-WBRT is not standard of care for PCI [7]. For optimized tumor control through WBRT, about 80 % of ROs applied a simultaneous integrated boost (SIB) on the metastases. This observation is in concordance with findings by Keit et al. [22], which identified Germany as the country using SIB the most in their international comparison. This possibly reflects the SIB concept being applied in the German HIPPORAD trial together with hippocampal sparing [28], [29]. It is important to mention that intracerebral tumor control is crucial for survival, but also for maintaining cognitive function [30], [31], [32].

To date, memantine is rarely prescribed in German-speaking countries (only 14 % of ROs using memantine), very much in contrast to both American guideline recommendations and American usage patterns (80 % using memantine) [17]. The substantial difference in memantine prescription underlines that recent clinical trials might have already significantly altered the therapeutic landscape of BM in the US, while German-speaking ROs currently remain hesitant to use memantine for clinical routine treatments. The most frequently mentioned reason for not using the drug was inexperience in using the drug (51 %), followed by limitations of currently available evidence (29 %). Further skepticism regarding memantine may arise from the necessary off-label use (17 %), as coverage by health insurances is unclear in this circumstance. In the US, changes in reimbursement policies might have already facilitated the widespread prescription of the drug. Concerns about side effects appear to play a minor role for German-speaking ROs (3 %), compared to ROs in the US (22.4  %) [17]. Further increasing pre-clinical and clinical evidence regarding the mechanism of action of memantine may gradually alter current opinions [33].

Interestingly, from concordance analyses, it appears that some centers make more use of neuroprotective strategies in general − regardless of technique − than others. It could be speculated that the knowledge of the three major clinical trials [10], [11], [13] plays a key role regarding this aspect, as it was shown in the US that those ROs familiar with the trials make use of neuroprotective techniques substantially more often [17]. Since 35 % of ROs in the US stated that the survey by Jairam et al. [17] increased their awareness about neuroprotective strategies and 23 % claimed that it will influence their practice, surveys like ours have the potential to change treatment patterns in Europe as well. From a patient’s perspective, the variation of use of different techniques at different centers is somewhat problematic. If the evidence allows for use of different techniques and if the capacity for all these techniques is given at a center, the patient’s choice should play a relevant part in an educated shared decision-making process.

It is important to note that our study has some limitations. While only about 20 % of all centers in German-speaking countries participated in completion of this survey, survey data were provided by most university hospitals; thus for this large segment of the healthcare system, it can be assumed that data are representative of the clinical workflow. The comparative analysis between different centers was limited by the relatively small sample size but comparable to the last German analysis (24 % of centers) [23]. As a further potential limitation, a possible tendency for ROs using neuroprotection to complete this survey must be taken into account. Further, we did not inquire for the available resources in each center. Of course, not just the ROs’ view on individual treatment techniques, but also the availability of new devices and technical and staff capacities are crucial for the implementation of novel treatments [34], [35]​.

It can be assumed that our findings cannot directly be transferred to the patterns of care in other European countries, as resources and healthcare systems substantially differ. Differences between Germany and its German-speaking neighboring countries could already be observed in our study, but a greater sample size is required to obtain a better understanding.

Our survey demonstrates that neuroprotective strategies are not yet implemented as standard procedures in daily clinical practice in German-speaking countries. There is substantial heterogeneity in general treatment of BM, e.g., regarding use of prognostic scores or WBRT techniques. While use of hippocampal sparing is increasing, most ROs remain hesitant to prescribe concomitant memantine. For most ROs in German-speaking countries, WBRT is still the treatment of choice for patients with more than 5 BM. Updated guidelines, in-depth education about clinical trial results and the publication of new data could aid the establishment of uniform neuroprotective standards of care for affected patients.

## Funding

This work did not receive any funding. Alexander Rühle was supported by a Clinician Scientist Program of the Medical Faculty of the University of Leipzig.

## CRediT authorship contribution statement

**N. Gleim:** Conceptualization, Data curation, Formal analysis, Investigation, Methodology, Writing – original draft, Writing – review & editing. **A. Rühle:** Resources, Writing – review & editing. **S. Heider:** Resources, Writing – review & editing. **F. Nägler:** Resources, Writing – review & editing. **F.A. Giordano:** Writing – review & editing. **S.E. Combs:** Writing – review & editing. **J. Becker:** Writing – review & editing. **M. Niyazi:** Writing – review & editing. **A.L. Grosu:** Writing – review & editing. **N.H. Nicolay:** Conceptualization, Data curation, Methodology, Project administration, Resources, Supervision, Writing – original draft, Writing – review & editing. **C. Seidel:** Conceptualization, Data curation, Formal analysis, Investigation, Methodology, Project administration, Resources, Supervision, Writing – original draft, Writing – review & editing.

## Data Availability

The dataset generated during the current study is available from the corresponding author on reasonable request.
