# Supplementary material for: Neuroprotection in radiotherapy of brain metastases: A pattern-of-care analysis in Germany, Austria and Switzerland by the German Society for radiation Oncology − working group Neuro-Radio-Oncology (DEGRO AG-NRO)
Source: Clin Transl Radiat Oncol. 2024 Apr 20;47:100783. doi: 10.1016/j.ctro.2024.100783 (PMC11063589; doi:10.1016/j.ctro.2024.100783)
Supplement: Supplementary Data 1 [file mmc1.docx]

Supplementary Material

Title: Neuroprotection in radiotherapy of brain metastases: a pattern-of-care analysis in Germany, Austria and Switzerland by the German Society for Radiation Oncology - Working Group Neuro-Radio-Oncology (DEGRO AG-NRO)

Authors: N. Gleim, A. Rühle, S. Heider, F. Nägler, F. A. Giordano, S. E. Combs, J. Becker, M. Niyazi, A. L. Grosu, N. H. Nicolay, C. Seidel

Corresponding Author: Clemens Seidel, Department of Radiotherapy and Radiation Oncology, University Hospital Leipzig, Stephanstraße 9a, Leipzig, Germany; [clemens.seidel@medizin.uni-leipzig.de](mailto:clemens.seidel@medizin.uni-leipzig.de)

| **Basic demographic data** | **n** | **%** |
| --- | --- | --- |
| **Type of radiotherapy facility** |  |  |
| University hospital | 24 | 30.8% |
| Non-university hospital | 24 | 30.8% |
| Outpatient center | 30 | 38.4% |
| **Country** |  |  |
| Germany | 70 | 89.8% |
| Austria | 4 | 5.1% |
| Switzerland | 4 | 5.1% |
| **Treated patients with BM per year** |  |  |
| < 10 | 0 | 0.0% |
| 10-50 | 32 | 41.6% |
| 51-100 | 28 | 36.4% |
| > 100 | 17 | 22.0% |
| **Leading position in RT facility** |  |  |
| Yes | 41 | 53.2% |
| No | 36 | 46.8% |
| **Treatment and follow-up patterns** | **n** | **%** |
| **Imaging follow-up organized by** |  |  |
| Radiotherapy | 37 | 48.1% |
| Oncology | 49 | 63.6% |
| Neurology | 3 | 3.9% |
| Neurosurgery | 11 | 14.3% |
| Interdisciplinary | 8 | 10.4% |
| **Clinical follow-up organized by** |  |  |
| Radiotherapy | 44 | 57.1% |
| Oncology | 52 | 67.5% |
| Neurology | 1 | 1.3% |
| Neurosurgery | 12 | 15.6% |
| Interdisciplinary | 8 | 10.4% |
| **Routine use of cognitive tests** |  |  |
| Prior to radiotherapy | 11 | 14.5% |
| Upon completion of radiotherapy | 1 | 1.3% |
| During follow-up | 11 | 14.5% |
| None | 53 | 69.7% |
| **Used cognitive tests** |  |  |
| Mini Mental Status Test (MMST) | 21 | 29.6% |
| Controlled Oral Word Association Test (COWA) | 2 | 2.8% |
| Demtect | 2 | 2.8% |
| Trail Making Test | 2 | 2.8% |
| Hamilton Verbal Learning Test (HVLT) | 1 | 1.4% |
| Alternative | 6 | 8.4% |
| None | 45 | 63.4% |
| **Use of prognostic scores regarded as** |  |  |
| Very important | 8 | 10.4% |
| Important | 33 | 42.9% |
| Rather unimportant | 30 | 38.9% |
| Not important | 6 | 7.8% |
| **Used prognostic scores** |  |  |
| GPA score | 12 | 15.8% |
| dsGPA score | 11 | 14.5% |
| RPA score | 17 | 22.2% |
| Other | 3 | 3.9% |
| None | 33 | 43.4% |
| **Technical aspects of WBRT** | **n** | **%** |
| **WBRT dose concept** |  |  |
| 3 Gy single, 30 Gy total dose | 54 | 70.1% |
| 2 Gy single, 40 Gy total dose | 5 | 6.5% |
| 4 Gy single, 20 Gy total dose | 3 | 3.9% |
| Alternative | 15 | 19.5% |
| **WBRT preferred over SRT starting from** |  |  |
| 1 BM | 0 | 0% |
| 2-3 BM | 1 | 1.3% |
| 4-5 BM | 17 | 21.8% |
| 6-10 BM | 36 | 46.2% |
| > 10 BM | 19 | 24.4% |
| Alternative | 5 | 6.4% |
| **Use of boost to BM** |  |  |
| Regularly | 12 | 15.4% |
| Frequently (≥ 50% of cases) | 26 | 33.3% |
| Occasionally (< 50% of cases) | 36 | 46.2% |
| Never | 4 | 5.1% |
| **Criteria for boost together with WBRT** |  |  |
| Size | 66 | 89.2% |
| General condition | 56 | 75.7% |
| Histology | 44 | 59.5% |
| Location | 43 | 58.1% |
| Age | 25 | 33.8% |
| Other | 11 | 14.5% |
| **Boost performed as** |  |  |
| Simultaneous integrated boost | 62 | 80.5% |
| Sequential | 27 | 35.1% |
| Stereotactic | 25 | 32.5% |
| None | 4 | 5.2% |
| **Hippocampal sparing** | **n** | **%** |
| **Use of hippocampal sparing with WBRT** |  |  |
| Regularly for therapy | 19 | 26.0% |
| Regularly for prophylaxis in SCLC | 27 | 36.9% |
| Occasionally for therapy | 35 | 47.9% |
| Occasionally for prophylaxis | 15 | 20.5% |
| Rarely / Not at all | 11 | 15.1% |
| **Use of HS in patients with BM and indication for WBRT** |  |  |
| Never | 8 | 10.9% |
| In < 10% of cases | 25 | 34.2% |
| In 10-49% of cases | 23 | 31.5% |
| In 50-80% of cases | 12 | 16.4% |
| In > 80% of cases | 5 | 6.8% |
| **Reasons for not applying therapeutic HS-WBRT** |  |  |
| CNS lymphoma | 45 | 63.3% |
| Visible adherent meningeosis foci | 45 | 63.3% |
| Estimated life expectancy < 6 months | 40 | 56.5% |
| Multiple small BM without hippocampal involvement | 33 | 46.5% |
| Karnofsky score < 60% | 33 | 46.5% |
| BM in SCLC | 26 | 36.6% |
| Age > 80 years | 21 | 29.6% |
| Alternative | 4 | 5.6% |
| **Dose parameters for HS-WBRT (open question)** |  |  |
| ***D_max_*** |  |  |
| ≤ 16 Gy | 7 | 29.2% |
| ≤ 17 Gy | 5 | 20.8% |
| Not known | 3 | 12.5% |
| Other | 9 | 37.5% |
| ***D_mean_*** |  |  |
| 9 Gy | 3 | 15.0% |
| 10 Gy | 4 | 20.0% |
| Not known | 3 | 15.0% |
| Other | 10 | 50.0% |
| ***D_100%_*** |  |  |
| ≤ 9 Gy | 7 | 46.7% |
| ≤ 10 Gy | 2 | 13.3% |
| Not known | 3 | 20.0% |
| Other | 3 | 20.0% |
| ***D_40%_*** |  |  |
| 7.5 Gy | 1 | 16.7% |
| Not known | 3 | 50.0% |
| Other | 2 | 33.3% |
| ***Other dose parameters*** |  |  |
| D_2%_ < 9 Gy | 1 | 8.3% |
| D_2%_ < 16 Gy | 1 | 8.3% |
| D_2%_ < 17 Gy | 2 | 16.7% |
| D_98%_ < 9 Gy | 3 | 25.0% |
| D_98%_ < 17 Gy | 1 | 8.3% |
| Other | 4 | 33.3% |
| **Use of HS if one hippocampus is affected by BM** |  |  |
| Yes | 30 | 41.7% |
| No | 42 | 58.3% |
| **Memantine** | **n** | % |
| **Used neuroprotective drugs with WBRT** |  |  |
| Memantine | 10 | 13.9% |
| Cortisone | 1 | 1.4% |
| None | 61 | 84.7% |
| **Use of memantine with therapeutic WBRT without HS** |  |  |
| Never | 60 | 85.7% |
| In < 10% of cases | 5 | 7.1% |
| In 10-49% of cases | 4 | 5.7% |
| In 50-80% of cases | 1 | 1.4% |
| In > 80% of cases | 0 | 0.0% |
| **Use of memantine with therapeutic WBRT with HS** |  |  |
| Never | 62 | 87.3% |
| In < 10% of cases | 4 | 5.6% |
| In 10-49% of cases | 3 | 4.2% |
| In 50-80% of cases | 1 | 1.4% |
| In > 80% of cases | 1 | 1.4% |
| **Use of memantine with prophylactic WBRT without HS** |  |  |
| Never | 63 | 88.7% |
| In < 10% of cases | 3 | 4.2% |
| In 10-49% of cases | 4 | 5.6% |
| In 50-80% of cases | 1 | 1.4% |
| In > 80% of cases | 0 | 0.0% |
| **Use of memantine with prophylactic WBRT with HS** |  |  |
| Never | 64 | 91.4% |
| In < 10% of cases | 1 | 1.4% |
| In 10-49% of cases | 3 | 4.3% |
| In 50-80% of cases | 1 | 1.4% |
| In > 80% of cases | 1 | 1.4% |
| **Personal view on memantine** |  |  |
| Advantageous | 13 | 21.7% |
| Without advantage | 44 | 73.3% |
| Rather disadvantageous | 3 | 5.0% |
| **Reasons for not prescribing memantine** |  |  |
| Lack of experience with the drug | 30 | 50.8% |
| Insufficient available evidence | 17 | 28.8% |
| Problems with reimbursement due to off-label use | 10 | 16.9% |
| Concerns about side effects | 2 | 3.4% |
| **Prescription of memantine via** |  |  |
| Health insurance after reimbursement request | 1 | 1.4% |
| Health insurance without reimbursement request | 3 | 4.3% |
| Self-pay with informed consent | 4 | 5.7% |
| Done by another specialist | 2 | 2.9% |
| Not performed | 60 | 85.7% |
| **Prescription period** |  |  |
| 3 months | 2 | 20.0% |
| 6 months | 7 | 70.0% |
| Without time limitation | 1 | 10.0% |

***Supplementary Table 1*** *Detailed questionnaires*. *Percentage values are shown relative to number of responding radiation oncologists. Abbreviations: BM brain metastases, RT radiotherapy, GPA graded prognostic assessment, dsGPA disease-specific GPA, RPA recursive partitioning analysis, WBRT whole brain radiotherapy, HS hippocampal sparing, SCLC small cell lung cancer.*
